# Evidence of Hepatitis B Virus Infection in Cancer and Noncancer Stem Cells Associated with Human Hepatocellular Carcinoma

**DOI:** 10.1155/2016/8931591

**Published:** 2016-03-29

**Authors:** Gerald Y. Minuk, Wendy Bautista, Julianne Klein

**Affiliations:** ^1^Section of Hepatology, Department of Medicine, University of Manitoba, Winnipeg, MB, Canada R3E 3P4; ^2^Department of Pathology, University of Manitoba, Winnipeg, MB, Canada R3E 3P4

## Abstract

Both the hepatitis B virus (HBV) and cancer stem cells (CSCs) have been independently implicated in the pathogenesis of hepatocellular carcinoma (HCC). To date, there have been no reports describing HBV infection within CSCs. In this report we describe HBV core (HBcAg) and HBx protein expression within CSCs associated with human HCC. HBV markers were also identified in nonmalignant stem cells present in adjacent nontumor tissue. These findings provide new insights into the pathogenesis of HBV-induced HCC and are potentially relevant to the treatment of both HCC and chronic HBV.

## 1. Introduction

Hepatocellular carcinoma (HCC) is the fifth most common cancer and third most common cause of cancer-related death in the world today [1]. HCC is particularly common in Southeast Asia and Sub-Saharan Africa where prevalences of hepatitis B virus (HBV) infection are highest.

Although HBV is considered an oncogenic virus, the mechanisms whereby it contributes to the development and/or growth of HCC remain to be fully elucidated [2]. Previous studies have indicated that HBx protein, a potent transcriptional transactivator, plays an important role in HCC carcinogenesis [3]. Hepatitis B core (HBcAg) may also be of pathophysiologic importance as the expression of this protein serves as a target for immune mediated chronic inflammation and eventually the development of cirrhosis, a premalignant condition [3]. HBcAg expression within hepatocytes also serves as a robust indicator of HBV infection [3].

Cancer stem cells (CSCs) represent a small subpopulation of malignant embryonic/progenitor cells that contribute to the development, growth, and metastasis of HCC [4]. CSCs can be identified (but not distinguished from nonmalignant stem cells) by their expression of various “stemness” markers including epithelial cell adhesion molecule (EpCAM), Oct-4, Nanog, and Notch and their presence within the tumor tissue compartment [5].

Despite well documented independent associations between HBV and HCC and CSCs and HCC and reports of increased HBx expression in HCCs with a high prevalence of CSCs, there have been no reports describing HBV markers within CSCs associated with human HCC [6].

## 2. Case Report

A 74-year-old Asian male with chronic HBV underwent surgical resection of a 9 cm HCC. The patient had not received antiviral therapy or chemotherapy or undergone prior ablative procedures. Processed sections of the specimen were deparaffinized, washed with buffer, and incubated overnight with commercially available primary monoclonal murine or rabbit antibodies to HBcAg, HBx, and the stem cell markers: EpCAM, Oct-4, Nanog, and Notch. Sections were then washed and incubated with donkey anti-mouse or anti-rabbit secondary antibodies prior to counterstaining the nuclei with DAPI.

**Figure 1:**
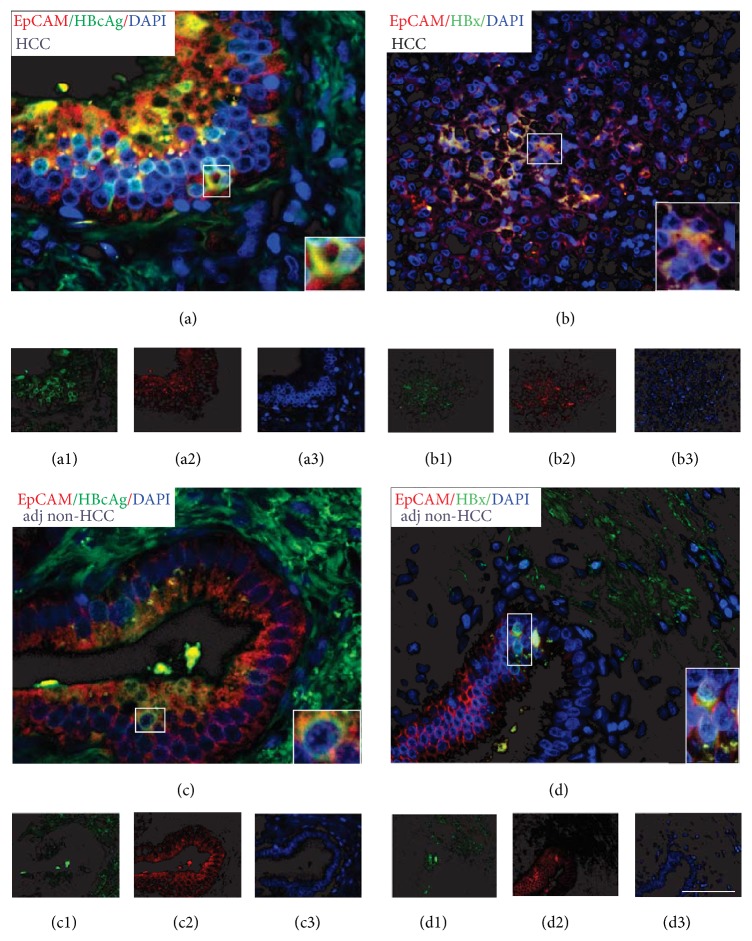
Immunofluorescence and confocal images of hepatitis B virus core antigen (HBcAg) and hepatitis B virus x protein (HBx) and the stem cell marker epithelial cell adhesion molecule (EpCAM) in human hepatocellular carcinoma (HCC) and adjacent nontumor tissues. Panel (a) overlay of HBcAg (red) and EpCAM (green) positive cells and DAP1 nuclei counterstaining (blue) in HCC tissue (panels (a1)–(a3), resp.). Insert: confocal image of HBcAg and EpCAM positive staining cell. Panel (b): overlay of HBx (red) and EpCAM (green) positive cells and DAP1 nuclei counterstaining (blue) in HCC tissue (panels (b1)–(b3), resp.). Insert: confocal image of HBx and EpCAM positive staining cell. Panel (c): staining of adjacent nontumor tissue for the same proteins described in panel (a). EpCAM positive staining cells were located in close proximity to the portal tracts of the liver lobule. Panel (d): staining of adjacent nontumor tissue for the same proteins described in panel (b). Magnification 40x and 60x (inserts).

As shown in Figure 1 for EpCAM staining, CSCs were abundant throughout the HCC and coexpressed both HBcAg (panel (a)) and HBx (panel (b)). The same results were obtained with Oct-4, Nanog, and Notch staining (data not shown). As predicted, stem cells were also present (albeit to a lesser extent than in HCC) in adjacent nonneoplastic liver, predominantly in close proximity to portal tracts where nonmalignant stem cells are known to be located. These cells also coexpressed HBcAg (panel (c)) and HBx (panel (d)) proteins. Neither HBcAg nor HBx were expressed in stem cells present within tumor or adjacent nontumor tissues from an HBV negative patient with HCC and another HBV negative patient with hepatocellular adenoma (data not shown).

## 3. Discussion

The mechanisms whereby HBV contributes to the development of HCC remain to be fully elucidated. During the course of infection, viral genome exists in both episomal and integrated form, resulting in the transcription of viral proteins including HBcAg and HBx [3]. While HBcAg is the principal target for immune mediated cytolysis and is often employed as a marker of HBV infection within cells, HBx may be more relevant to the pathogenesis of HCC in that it possesses transcriptional transregulatory properties that include modulating the transcriptional activation of p53, interruption of apoptosis, inhibiting proteasomal degradation of growth regulatory proteins, and stimulating cellular kinases that alter signal transduction [7].

A number of studies have implicated CSCs in the pathogenesis and course of HCC [8, 9]. Thus, interfering with the development of CSCs and/or eradicating these cells from the tumor population would be expected to have a favorable impact on the incidence and treatment of HCC, respectively.

In two previous studies addressing a possible association between HBV and CSCs, Arzumanyan et al. described transfection of “mature” HepG2 cells with HBx and the subsequent appearance of “stemness” as reflected by EpCAM, Oct-04, Nanog, HIf-4, and B-catenin expression [10]. The authors also analyzed HCC and adjacent nontumor tissues for HBx and stem cell markers and found positive staining in both tissues (more so in adjacent than tumor tissue). However, it was not stated as to whether HBx expression was present in the same cells expressing “stemness” markers. Similar findings were described by Wang et al. where HBx expression levels correlated with the prevalence of CSCs but merging of costained tissues was not performed. Thus, whether expression of HBV and “stemness” markers was occurring in the same cell population remains unclear [6].

The present report is the first to describe a direct association between HBV and CSCs as well as HBV and nonmalignant liver stem cells in humans. This association raises interesting questions that warrant further research. For example, does HBV infect CSCs directly or does the virus infect nonmalignant stem cells resulting in their malignant transformation? Alternatively, does HBV infect mature hepatocytes causing them to dedifferentiate into a stem cell lineage as proposed by Arzumanyan et al. and Holczbauer et al. [10, 11]? If HBV does infect stem cells (malignant or nonmalignant), what receptor does the virus utilize to gain entry to these cells? Also to be determined is whether the presence of HBV within nonmalignant stem cells contributes, via clonal expansion of infected stem cells, to the abundance of HBV infected hepatocytes within the liver (i.e., beyond the proposed paracrine viral infection of adjacent mature hepatocytes). From a treatment perspective, it will be important to determine whether the presence of HBV markers within CSCs could serve as a target for CSC eradication and thereby HCC treatment. Also important is the question whether antiviral therapy will clear HBV from CSCs or will the upregulation of multidrug resistant proteins that are a feature of CSCs prevent such clearance [12]? Finally, does antiviral therapy clear HBV from nonmalignant stem cells and if not, could these cells represent a reservoir for the frequent relapses that occur following cessation of antiviral treatment?

In conclusion, to our knowledge, this is the first report that describes HBV marker expression within CSCs associated with human HCC and nonmalignant stem cells associated with adjacent non-tumor tissue. In addition to providing important insights into the pathogenesis of HCC, the natural history of HBV, and new target cell populations for HBV treatment, these findings should encourage the search for other oncogenic viruses in other CSC associated cancers.
